# Valorization of Isabella Grape (*Vitis labrusca* L.) Pomace Through the Recovery of Nutraceuticals by Sequential Green Extraction Technologies

**DOI:** 10.3390/foods15010054

**Published:** 2025-12-24

**Authors:** Jhonattan Sánchez Sánchez, Fabián Parada-Alfonso, Henry I. Castro-Vargas

**Affiliations:** 1 Grupo de Investigación en Química de Alimentos (GIQA), Departamento de Química, Facultad de Ciencias, Universidad Nacional de Colombia, Carrera 30 No 45-03, Bogotá 110911, Colombia; jsanchezs@unal.edu.co (J.S.S.); fparadaa@unal.edu.co (F.P.-A.); 2 Grupo de Investigación en Electroquímica y Medio Ambiente (GIEMA/CICBA), Departamento de Ciencias Naturales, Extractas y Estadística, Facultad de Ciencias, Universidad Santiago de Cali, Campus Pampalinda, Calle 5 No 62-00, Santiago de Cali 760035, Colombia

**Keywords:** Isabella grape pomace, supercritical fluid extraction, pressurized liquid extraction, nutraceuticals, tocopherols, phenolic compounds, biorefinery, antioxidant activity

## Abstract

Isabella grape pomace (IGP) the primary by-product of Colombia’s winemaking industry, represents a promising source of nutraceuticals with potential uses in the food industry. This study developed a sequential green extraction process to recover nutraceutical from IGP. The approach integrated supercritical fluid extraction with CO_2_ (SFE-CO_2_) to obtain lipophilic compounds, followed by SFE with ethanol/water as co-solvent (SFE-CO_2_/EtOH:H_2_O) for medium-polarity phenolics, and pressurized liquid extraction (PLE) with EtOH:H_2_O to recover the polar phenolic-rich fraction. The extraction parameters were optimized using response surface methodology, and optimal conditions were identified: SFE-CO_2_ at 31.7 MPa/58.9 °C yielded 6.95% extract rich in linoleic acid (65.5%) and α-tocopherol (107.2 mg/kg); SFE-CO_2_/EtOH:H_2_O with 15% of co-solvent produced extracts with high phenolic content (105.35 mg GAE/g) and antioxidant activity (0.18 mmol TE/g); while PLE at 58.91% of EtOH/107.98 °C achieved notable recovery of flavonoids (757.18 mg QE/g), anthocyanins(1508 μg MAE/g) and condensed tannins (258.39 mg ECE/g), with potent antioxidant capacity (130.40 mmol TE/g). The sequential process demonstrated synergistic effects, with a total cumulative yield of 41.08% and phenolic recovery of 349.89 mg GAE/g extract. This approach offers a sustainable biorefinery approach for transforming IGP into high-value nutraceutical ingredients.

## 1. Introduction

The Isabella grape (*Vitis labrusca* L.), also known as Isabel, raspberry grape, or black raspberry grape, is a natural hybrid between *Vitis vinifera* (European vid) and *Vitis labrusca* (American vid). Native from North America, its cultivation has spread throughout South America with a significant presence in countries such as Brazil, Argentina, Chile, and Colombia where it is primarily used in the production of artisanal wines [1]. This particular cultivar is recognized for its high content of valuable bioactive compounds, including organic acids (e.g., tartaric, citric, pyruvic, and shikimic), vitamins (e.g., B_1_ and B_2_), and, most notably, a diverse profile of phenolic compounds. Furthermore, the seeds of Isabella grape, a valuable fraction of the pomace, serve as a rich source of protein, lipids, and the renowned resveratrol [2].

The cultivation of Isabella grape is predominantly concentrated in South America. Colombia stands as the leading producer, followed by Brazil and Argentina. Notably, Colombian production alone has ranged between 30 and 35 thousand tons per year over the last five years, with the entirety of this harvest being dedicated to wine manufacturing [3]. It is estimated that at least 20% of Isabella grape processed in the wine production becomes waste; the primary solid residue is the pomace, a by-product composed of grape skins, seeds, and occasionally stems [4]. Data from the Colombian wine industry, particularly from companies like Casa Grajales S.A., indicate that the average annual generation of grape pomace from Isabella grapes reached approximately 8000 tons between 2021 and 2024 [1,3]. Although wine industries have sought alternatives for utilizing this waste (e.g., composting), a significant portion continues to be disposed of in landfills, where it contributes to environmental pollution.

Currently, it is well established that agro-industrial wastes are a rich source of high-value compounds with potential applications in the food and cosmetic industries [5,6]. Specifically, wine industry by-products are recognized as a notable reservoir of bioactive compounds, including polyunsaturated fatty acids (PUFAs), tocopherols, tocotrienols, and a wide array of phenolic compounds [7]. There is now extensive scientific evidence demonstrating the nutraceutical properties of the bioactive substances present in extracts, fractions, and formulations derived from wine industry waste. Among these, their antioxidant, antibacterial, anti-inflammatory, anticancer, hypoglycemic, hypolipidemic, cardioprotective, hepatoprotective, and neuroprotective properties are particularly notable [8,9]. While the potential of wine pomace is well-documented, research on Isabella grape pomace (IGP) specifically lags behind. Although a few studies have characterized IGP extracts obtained by conventional methods [10,11], significant gaps persist in the literature. Most notably, the application of efficient and sustainable green extraction techniques—such as supercritical fluid extraction (SFE) and pressurized liquid extraction (PLE)—to IGP, and their strategic integration into a sequential process for the fractionated recovery of nutraceuticals, remains largely unexplored.

The valorization of agro-food waste requires the implementation of innovative and environmentally friendly technologies. These technologies must be designed to obtain products with optimal qualities—including good bioactive properties, yield, and efficiency at low cost—while ensuring a low or negligible environmental impact. Furthermore, for applications in the food, and cosmetic industries, the extraction solvents must be classified as Generally Recognized As Safe (GRAS) to ensure the absence of toxic residues in the final product. In this context, SFE and PLE are recognized as green extraction technologies with viable applications in the recovery of nutraceuticals from various matrices. SFE utilizes fluids above their critical point (e.g., CO_2_ at >31 °C and >7.4 MPa), where they exhibit liquid-like density and gas-like diffusivity, enabling efficient penetration into solid matrices and selective solvation of target compounds. While PLE, also known as accelerated solvent extraction, employs solvents at high pressures (typically 5–20 MPa) and temperatures (50–200 °C) above their normal boiling point. This combination increases solubility and mass transfer rates while decreasing solvent viscosity and surface tension, leading to faster and more efficient extractions compared to techniques at atmospheric pressure [12,13,14,15]. These techniques meet the demands for sustainability, efficiency, and safety previously outlined. Notably, SFE has advanced beyond the laboratory scale and is now successfully implemented at an industrial level for the recovery of a wide range of high-value products, including antioxidants, oils, aromas, pigments, and flavorings [16].

While SFE is highly effective for the extraction of non-polar compounds (e.g., PUFAs and tocopherols), PLE demonstrates good performance for the recovery of medium to polar compounds, particularly phenolics [17]. The sequential combination of these two technologies represents a promising and integrated approach for the comprehensive valorization of complex matrices, allowing for the fractionation and recovery of a broader spectrum of bioactive compounds based on their polarity. This strategy has been successfully applied to other agro-industrial residues [13,18,19,20], but its potential for Isabella grape pomace remains unexplored.

The preceding background demonstrates that IGP is a potential source of nutraceuticals and that SFE and PLE are appropriate techniques for their recovery. In the context of this work, the term “nutraceuticals” refers to bioactive compounds that exhibit proven or potential health-promoting properties beyond basic nutrition, such as antioxidant, anti-inflammatory, or cardioprotective activities. These include, but are not limited to, the polyunsaturated fatty acids, tocopherols, and phenolic compounds (e.g., flavonoids, anthocyanins, tannins) targeted in this sequential extraction process. Therefore, this study aims to develop a sequential green extraction process to recover nutraceuticals from IGP as an alternative for its comprehensive valorization. The approach involves an initial SFE stage using neat carbon dioxide (CO_2_) to recover the lipophilic fraction (rich in PUFAs and tocopherols) followed by a SFE stage with CO_2_ added with an ethanol/water mixture as co-solvent (CO_2_/EtOH:H_2_O), and a PLE stage using EtOH:H_2_O mixtures to extract the medium to polar phenolic-rich fraction, respectively. The obtained fractions were characterized in terms of global yield, chemical composition (PUFAs and Tocopherols), phenolics content (flavonoids, anthocyanins, and condensed tannins), and antioxidant activity. This work proposes a sustainable biorefinery model for this waste, transforming it into high-value nutraceutical ingredients.

## 2. Materials and Methods

### 2.1. Isabella Grape Pomace and Pretreatment

IGP, comprising a mixture of skins and seeds, was supplied by Casa Grajales S.A. (La Unión, Valle del Cauca, Colombia), obtained as a solid residue from wine manufacturing. Upon reception, approximately 100 kg of fresh pomace was dried at ambient temperature (15 to 18 °C) for two days to prevent the thermal degradation of heat-sensitive bioactive compounds. The dried pomace was then ground using a knife mill. The powdered material was sieved using US Standard size sieves, and the fraction with a particle size between 0.210 and 0.420 mm (+70/−40 mesh) was selected for all extraction experiments. The homogenized sample was stored in sealed high-density polyethylene bags at 4 °C in a dark and dry place until use. The moisture content of the dried and ground pomace was determined to be <10% (*w*/*w*).

### 2.2. Sequential Green Extraction Process

**Figure 1:**
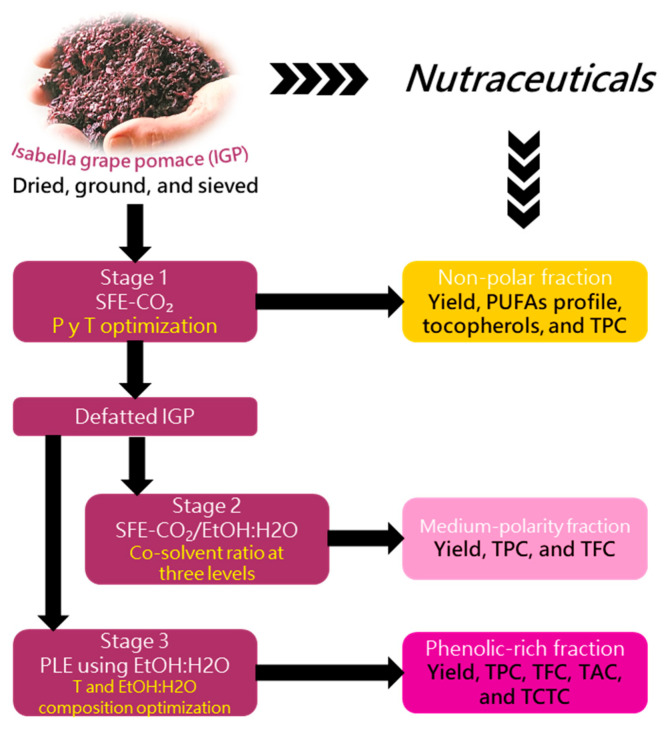
General scheme for the nutraceuticals recovery from Isabella grape pomace using a sequential green extraction process.

The recovery of nutraceuticals from IGP was accomplished through a sequential green extraction process, as outlined in Figure 1. The initial step consisted of SFE with pure CO_2_ (SFE-CO_2_); the operating pressure and temperature were optimized to maximize the yield of the non-polar fraction and to produce a defatted pomace substrate. This defatted IGP was then processed in a second SFE stage employing CO_2_ added with an ethanol/water mixture as co-solvent (SFE-CO_2_/EtOH:H_2_O), where the co-solvent ratio was changed to recover the medium-polarity fraction. Simultaneously, the defatted IGP was valorized through PLE using EtOH:H_2_O mixtures. For this stage, the solvent composition and temperature were optimized to efficiently extract the polar phenolic-rich fraction.

#### 2.2.1. Supercritical Fluid Extraction

**Figure 2:**
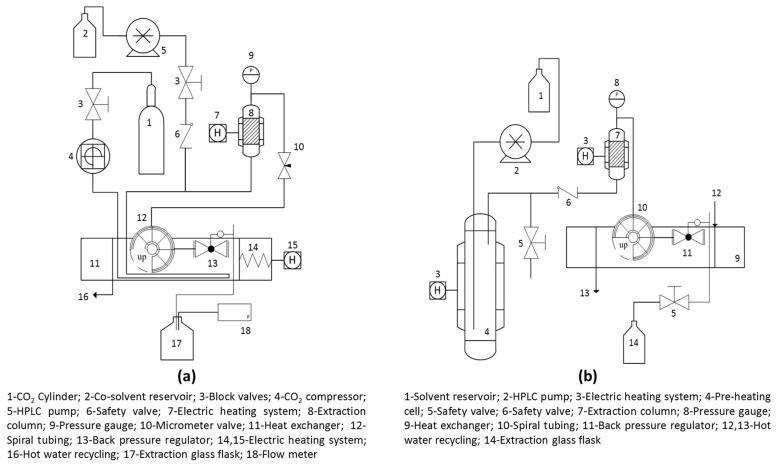
Schematic diagram of the extraction units employed: (**a**) Supercritical fluid extraction system and (**b**) pressurized liquid extraction system.

SFE experiments were performed using a dynamic extraction system schematized in Figure 2a. For each run, a fixed mass of 10.0 g of dried and ground IGP was placed inside the extraction column to form a fixed bed of particles. The remaining space in the extractor was filled with glass beads and cotton wool at the bottom and top to prevent channeling and ensure uniform solvent distribution. Then the extraction parameters (temperature, pressure, co-solvent percentage, and solvent flow rate) were adjusted. The extraction time for all experiments was established through preliminary kinetic assays. For SFE-CO_2_ experiments these assays were conducted at a constant temperature of 50 °C, a pressure of 25 MPa, and a CO_2_ flow rate of 0.36 ± 0.05 kg/h, while SFE-CO_2_/EtOH:H_2_O kinetic tests were performed at 58.9 °C, 31.7 MPa, 10% *w*/*w* of ethanol/water, and the same CO_2_ flow rate (0.36 ± 0.05 kg/h). Based on the kinetic curves (yield vs. time), the extraction time was set at 240 min for SFE-CO_2_ experiments and 330 min for SFE-CO_2_/EtOH:H_2_O experiments. These were determined to be sufficient to reach the diffusion-controlled period of the extraction process. All extractions were performed at the constant CO_2_ flow rate mentioned above. The mixture solvent/extract (e.g., CO_2_/extract or CO_2_/EtOH:H_2_O/extract) was separated to ambient pressure (101.3 kPa) and the extracts were collected in amber flasks. For the SFE-CO_2_/EtOH:H_2_O experiments, ethanol/water was removed under vacuum. All extracts were weighted in an analytical balance, then flushed with a nitrogen stream, sealed, and stored at −20 °C for further analyses. Finally, the extraction yield was calculated as the percentage of mass of extract related to the mass of IGP used (% *w*/*w* d.b.).

A rotable central composite design (RCCD), with α = 1.414, was applied for the optimization of SFE-CO_2_ stage. The independent variables were temperature (*T*: 40–60 °C) and pressure (*P*: 20–30 MPa); these ranges were chosen to balance solvent power (CO_2_ density), solute volatility, and the thermal stability of target compounds like tocopherols and PUFAs [12,16]. The response variables were extraction yield and total phenolic content (TPC). The design consisted of 12 experimental runs which were performed in randomized in order to minimize the effects of unexpected variability. The central point was replicated four times to estimate the experimental error. The complete design matrix, including the coded and actual levels of the variables, is presented in Table 1. The second order models were obtained according to Equation (1) to aim at explaining the *Y_n_* response variables as a function of the extraction parameters:

(1) $$Y_{n}=\beta_{0}\;+\;\beta_{1}T\;+\;\beta_{2}P\;+\;\beta_{12}\mathrm{TP}\;+\;\beta_{11}T^{2}+\beta_{22}P^{2}$$

where *β*_0_ is the intercept, *β*_1_ and *β*_2_ are linear regression coefficients, *β*_11_ and *β*_22_ are quadratic regression coefficients, and *β*_12_ is the interaction coefficient. The effect of each experimental parameter on the response variables was studied at 95% confidence level (*p* < 0.05). For each response, an empirical model was developed to correlate the independent variables. The adequacy of the developed models was tested by comparing the predicted results with the experimental values. Model accuracy was assessed using the correlation coefficient (R^2^) and analysis of variance (ANOVA). Additionally, multiple response optimization was performed by combining the experimental parameters to maximize the desirability function, thus determining the overall optimal extraction conditions for the SFE-CO_2_ stage.

A defatted IGP sample was prepared using the optimal extraction conditions obtained in the SFE-CO_2_ step. This biomass was then used to recover the medium-polarity fraction. The extractions were performed at a fixed temperature of 58.9 °C and pressure of 31.7 MPa, based on preliminary findings. An ethanol/water mixture (57:43 *v*/*v*) was used as the co-solvent. This specific mixture was selected based on previous reports indicating its high efficiency for the extraction of phenolic compounds from wine pomace [21]. The co-solvent percentage was evaluated at three levels: 5, 10, and 15% (*w*/*w*). All other parameters (extraction time of 330 min, CO_2_ flow rate of 0.36 ± 0.05 kg/h) were maintained constant as established previously. The extraction yield was calculated for all experiments, and the obtained extracts were further characterized in terms of TPC, total flavonoids content (TFC), and antioxidant activity.

#### 2.2.2. Pressurized Liquid Extraction

The defatted IGP, obtained from the SFE-CO_2_ stage under optimal conditions, was submitted to PLE using EtOH:H_2_O mixtures at different ratios and temperatures to recover the phenolics-rich fraction. All experiments were conducted in a dynamic mode using the extraction unit described in Figure 2b. The general extraction procedure was as follows: A fixed mass of 10.0 g of sample was loaded into the column to form a fixed bed. Defatted cotton was placed at the bottom and top of the bed to ensure uniform solvent distribution and prevent particle leakage. The system was sealed, and the desired EtOH:H_2_O solvent mixture was pumped through the fixed bed until it reached a fixed pressure of 10 MPa. The extraction temperature was then set and maintained, and solvent flow rate was kept constant at 0.3 kg/h for all experiments. The total extraction time for each run was set at 120 min; this time was established through kinetic assays, which were conducted at constant conditions of 10 MPa, 80 °C, and a solvent composition of 50:50 (*v*/*v*) EtOH:H_2_O. For all extracts the mixture solvent/extract was separated using high vacuum and freeze-drying, then these were weighted and yield was calculated (as % *w*/*w* d.b.). Finally, dry extracts were flushed with a nitrogen stream, sealed, and stored at −20 °C for further analyses.

A RCCD (α = 1.414) was used to optimize independent variables temperature (*T*: 60–100 °C) and solvent composition, expressed as the ethanol percentage (*C*: 40–60% *v*/*v*). Parameters ranges were optimized to maximize phenolic recovery while minimizing thermal degradation [17,22]. Extraction yield, TPC, TFC, total anthocyanin content (TAC), and total condensed tannins content (TCTC) were selected as response variables. The design plan consists of 12 randomized runs with four replicates at the central point. The complete design matrix is shown in Table 2. The quadratic model proposed for each response variable *Y_n_* was obtained according to Equation (2):

(2) $$Y_{n}=\beta_{0}\;+\;\beta_{1}T\;+\;\beta_{2}C\;+\;\beta_{12}\mathrm{TC}\;+\;\beta_{11}T^{2}+\beta_{22}C^{2}$$

where *β*_0_ is the intercept, *β*_1_ and *β*_2_ are linear regression coefficients, *β*_11_ and *β*_22_ are quadratic regression coefficients, and *β*_12_ is the interaction coefficient. The effect of each experimental parameter on the response variables was studied at 95% confidence level (*p* < 0.05). For testing the developed models, the predicted results and experimental values were compared. Model accuracy was assessed using the correlation coefficient (R^2^) and ANOVA. Finally, multiple response optimization was performed using the desirability function approach to identify the optimal PLE conditions for the simultaneous maximization of the target response variables.

### 2.3. Analysis of Nutraceuticals in SFE and PLE Extracts

#### 2.3.1. Determination of Tocopherols

The non-polar fraction obtained by SFE-CO_2_ under optimal conditions was submitted to analysis by gas chromatography coupled with mass spectrometry (GC-MS) in order to establish its tocopherols content. Prior to analysis, the extracts underwent a saponification using potassium hydroxide, followed by extraction of the unsaponifiable matter using diethyl ether. For GC-MS analysis an Agilent 7890A GCequipped with Agilent 5975 MSD (Palo Alto, CA, USA) and a HP-5MS (Agilent J&W 19091S-433, Santa Clara, CA, USA) column (30 m × 0.25 mm ID, 0.25 µm) was used. The injector temperature was set at 250 °C, and samples were injected in splitless mode. The transfer line was used at 280 °C. The GC oven temperature program was as follows: initial temperature of 200 °C held for 3 min, then ramped to 280 °C at a rate of 8 °C/min. Helium (99.999% purity) was used as the carrier gas at a constant flow rate of 1.0 mL/min. The mass spectrometer was operated in electron impact (EI) mode at 70 eV. The MS quadrupole and ion source temperatures were set at 150 °C and 250 °C, respectively. The mass spectrometer was set to scan mode in the mass range of 50–450 amu. Preliminary identification of tocopherols was performed by comparing their mass spectra with those from the NIST 2008 mass spectral library. The identifications were confirmed by comparing the retention times and mass spectra of the sample peaks with those of authentic external standards. Quantification was performed using external calibration curves. The results were expressed as milligrams of tocopherol per kg grams of extract (mg/kg extract). All analyses were conducted in triplicate.

#### 2.3.2. Determination of Fatty Acids Composition

The lipophilic fraction obtained by SFE-CO_2_ under optimal conditions was analyzed for its fatty acids content by GC-MS following the AOAC method (969.33, 1990) [23]. The extract was undergoing transesterification to obtain the fatty acid methyl esters (FAMEs). The GC-MS analysis was performed using the same equipment and column described in Section 2.3.1. The GC oven temperature program was as follows: initial temperature of 130 °C kept for 5 min, then ramped from 130 to 270 °C at a rate of 20 °C/min. The injector temperature was 250 °C, and the FAMEs sample injection was performed in split mode (1:170). Helium at 1.2 mL/min was used as carrier gas. The mass spectrometer was operated in EI mode at 70 eV, and the MS quadrupole and ion source temperatures were set at 150 °C and 250 °C, respectively. Data were acquired in full scan mode within a mass range of 50–450 amu. The identification of FAMEs obtained were made by comparing their mass spectra and retention times with the NIST 2008 mass spectral library reference standards. The results were expressed as the relative percentage of each fatty acid identified in the total FAME profile.

#### 2.3.3. Total Phenolic Content

The TPC in all extracts was determined by the Folin–Ciocalteu method following the procedure adapted from Castro-Vargas et al. [24] with slight modifications. Briefly, the dry extracts were dissolved in a mixture of ethyl acetate/ethanol (1:1) at final concentration range from 10 to 20 mg/mL. An aliquot of 20 μL of each extract solution was mixed with 1580 µL of distilled water and 100 µL of the Folin–Ciocalteu reagent. After 7 min, 300 µL of sodium carbonate solution (20% *w*/*v*) were added. Finally, the mixture was stirred and left to react in the dark and room temperature for 120 min. The absorbance at 765 nm (Thermo Scientific Evolution 600 UV/Vis, Waltham, MA, USA) was measured. The TPC was quantified using a standard calibration curve of gallic acid (concentration range: 50–500 μg/mL). The results are expressed as milligrams of gallic acid equivalents per gram of dry extract (mg GAE/g extract). All measurements were carried out in triplicate.

#### 2.3.4. Total Flavonoids Content

The total flavonoid content (TFC) of the extracts obtained from the SFE-CO_2_/EtOH:H_2_O and PLE stages was determined using the colorimetric method based on aluminum complexation, adapted from Chang et al. [25] with slight modifications. Briefly, dry extracts were dissolved in an ethanol/water (1:1, *v*/*v*) mixture to achieve final concentrations ranging from 10 to 20 mg/mL. Then a reaction mixture was prepared by combination of 200 µL of extract solution, 600 µL of ethanol, 40 μL of AlCl_3_ (10% *w*/*v*), 40 μL of sodium acetate (1 M), and 1120 μL of distillated water. The mixture vortexed was and keep under reaction at ambient temperature during 30 min. The absorbance of the solution was then measured at 415 nm using a Thermo Scientific Evolution 600 UV/V (Waltham, MA, USA) spectrophotometer. The TFC was quantified using a standard calibration curve of quercetin (concentration range: 10–100 µg/mL). The results are expressed as milligrams of quercetin equivalents per gram of dry extract (mg QE/g extract). All analyses were performed in triplicate.

#### 2.3.5. Total Anthocyanin Content

The TAC of the extracts obtained by PLE was determined using the pH differential method [26]. Briefly, each extract was diluted with a pH 1.0 potassium chloride buffer (0.025 M KCl) so that its absorbance at 520 nm fell within the range of 0.6 ± 0.2 AU. Using this same dilution factor, two separate aliquots were prepared: one diluted in the pH 1.0 KCl buffer and the other in a pH 4.5 sodium acetate buffer (0.4 M CH_3_COONa). The absorbance of each solution was then measured at both 520 nm and 700 nm against a blank of distilled water. The TAC results are expressed as micrograms of monomeric anthocyanin equivalents per gram of dry extract (µg MAE/g extract) established using as reference the cyaniding-3-glucoside. All analyses were performed in triplicate.

#### 2.3.6. Total Condensed Tannins Content

The TCTC was determined using the methylcellulose precipitation method [27]. Briefly, 200 µL of each ethanol/water extract solution was mixed with 200 µL of a 0.04% (*w*/*v*) methylcellulose solution and vortexed for 1 min. Then, 400 µL of a saturated ammonium sulfate solution was added, and the final volume was adjusted to 2 mL with deionized water. The solution was allowed to stand at room temperature for 10 min and then centrifuged for 5 min at 4000 rpm. The absorbance of the resulting supernatant was measured at 280 nm. The TCTC was quantified using a calibration curve prepared with (-)-epicatechin as the standard, with concentrations ranging from 10 to 250 mg/L. The results are expressed as milligrams of (-)-epicatechin equivalents per gram of dry extract (mg ECE/g extract). All analyses were performed in triplicate.

### 2.4. Antioxidant Activity (DPPH Radical Scavenging)

The DPPH scavenging activity was determined by following the procedure previously described by Castro-Vargas et al. [24]. Briefly, 1 mL of DPPH solution 0.1 M in ethanol, with initial absorbance measured at 517 nm (A_0_), was added with 50 μL of each extract solution. After 60 min, the final absorbance at 517 nm (A_f_) was measured. The scavenging percentage was determined as % = (A_0_ − A_f_)/A_0_ and compared against a standard calibration curve of Trolox (concentration range: 5–50 μM). The results are expressed as millimol of Trolox equivalents per gram of dry extract (mmol TE/g extract). All measurements were carried out in triplicate.

### 2.5. Statistical Analysis

The statistical differences in extraction yield, TPC, TFC, TAC, and TCTC were determined by means of ANOVA. Tukey test was employed for multiple comparisons. All the statistical analyses were developed using the software Statgraphics Centurion XVII with a confidence level of 95%.

## 3. Results and Discussion

### 3.1. Stage 1: Recovery of Non-Polar Fraction

The initial stage of the sequential process was focused on recovering the lipophilic fraction from the IGP using supercritical CO_2_. A rotatable central composite design was employed to optimize the process parameters (temperature and pressure) using the extraction yield and TPC as the response variables. Table 1 presents the extraction yields and TPC for non-polar extracts recovered from IGP. The highest yield (6.60%) was achieved at 30 MPa and 60 °C; this was followed by the yields obtained at 30 MPa and 40 °C (6.41%) and 30 MPa and 40 °C (6.22%). In contrast, the maximum TPC value of 7.05 ± 0.46 mg GAE/g extract was observed in the extract recovered at 32.1 MPa and 50 °C, while the second-highest TPC (6.48 ± 0.07 mg GAE/g extract) was recorded under the conditions of 30 MPa and 60 °C. The obtained yields are consistent with previous studies reporting values ranging from 5% to 20% for oil extraction from by-products of various grape varieties, including the Isabella cultivar [28,29]. A similar trend was observed for the TPC values, which were consistent with those reported for lipophilic fractions from other grape pomaces (0.010 to 10 mg GAE/g extract) [30].

The optimization of extraction parameters was performed by fitting the experimental data (Table 1) to a response surface model (RSM) using multiple regression analysis. This approach was employed to describe the effects of SFE-CO_2_ variables on the extraction yield and TPC of the extracts obtained from IGP. The experimental data were fitted to a second-order polynomial model. For the extraction yield, the resulting model equation (Equation (3)) was obtained with a coefficient of determination (R^2^) of 86.46%, an adjusted R^2^ of 75.17%, and a standard error of 0.46. The model was found to be adequate, as indicated by the high R^2^ value and a non-significant lack of fit (*p* = 0.12), confirming its satisfactory ability to explain the behavior of the extraction process.

(3) $$Yield=4.72-0.23T\;+0.56P\;+0.70\mathrm{TP}\;+0.72T^{2}+0.26P^{2}$$

**Figure 3:**
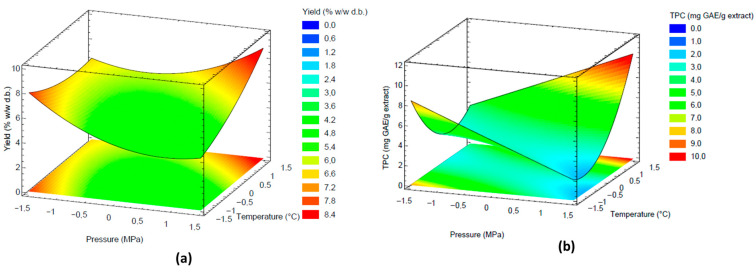
Response surface plots of the effects of SFE-CO_2_ parameters on (**a**) extraction yield and (**b**) TPC. Temperature and pressure coded levels are described in Table 1.

The Pareto Chart for yield and TPC are presented at Supplementary Material (Figure S1). According to the Pareto’s analysis, the parameters with the highest effect on the extraction yield were the quadratic coefficient of temperature (*p* = 0.008) and the linear coefficient of pressure (*p* = 0.014). In addition, the interaction between temperature and pressure showed also a significant effect (*p* = 0.024). The effects of temperature and pressure on the extraction yield are visualized in the response surface plot shown in Figure 3a. At high pressures, the yield increased with temperature. In contrast, at low pressures, the opposite effect was observed. In the first case, the observed behavior can be attributed to an increase in the vapor pressure of the solutes, whereas the second trend is primarily related to a reduction in the density of supercritical CO_2_. Furthermore, a positive effect of pressure on the yield was observed at medium to high temperature levels. This behavior is primarily attributed to the increase in supercritical CO_2_ density, which enhances its solvent power.

TPC data was fitted to the second-order model, and Equation (4) was obtained. The high values of R^2^ (94.74%) and adjusted R^2^ (90.36%), coupled with a non-significant lack of fit (*p* = 0.07), demonstrate that the regression model satisfactorily explains the effects of pressure and temperature on phenolic compound extraction using SFE-CO_2_. The Pareto Chart revealed that the quadratic coefficient of pressure (*p* = 0.0003), the interaction between temperature and pressure (*p* = 0.0009), and the linear coefficient of pressure (*p* = 0.0081) had highly significant effects on the phenolic compound recovery from IGP using supercritical CO_2_. These effects are evident in Figure 3b, which shows that the highest TPC values were obtained at pressures above 30 MPa and temperatures exceeding 50 °C.

(4) $$TPC=3.05+0.03T+0.65P+1.45\mathrm{TP}+0.01T^{2}+1.37P^{2}$$

The results indicate that SFE-CO_2_ employing high pressures (>30 MPa) and temperatures (>50 °C) simultaneously enhances the recovery of the lipophilic fraction from IGP, yielding favorable extraction efficiencies and phenolic contents. In order to establish the optimal conditions for SFE-CO_2_ stage, a multiple response optimization (MRO) was performed. Then, the optimum SFE-CO_2_ conditions (SFE-CO_2optimal_) were defined as 31.7 MPa and 58.9 °C with desirability function of 0.94. The MRO model predicted optimal values of 7.15% for extraction yield and 8.19 mg GAE/g extract for TPC. Experimental verification under SFE-CO_2optimal_ confirmed these predictions, yielding 6.95 ± 0.09% and 8.08 ± 0.21 mg GAE/g extract, respectively.

Chemical characterization of the SFE-CO_2_ optimal extract was performed to quantify its nutraceutical compounds, specifically fatty acids and tocopherols. The results are summarized in Table 3. The predominant nutraceuticals identified in the lipophilic fraction were linoleic acid (C18:2), oleic acid (C18:1), α-Tocopherol, and γ-Tocopherol. These results align with previous reports indicating that linoleic acid and α-Tocopherol are the main nutraceuticals in oils extracted from various grape by-products, including seeds, skins, and pomace [29]. In summary, stage 1 successfully established the optimal SFE-CO_2_ parameters for obtaining a nutraceutical-enriched lipophilic fraction from IGP, containing phenolic compounds, mono- and polyunsaturated fatty acids, and tocopherols.

### 3.2. Stage 2: Recovery of Medium-Polarity Fraction

Prior to the development of stage 2, a defatted IGP sample was prepared using the optimal conditions established in stage 1. This biomass was subsequently subjected to extraction using CO_2_ added with a mixture of ethanol/water as co-solvent (SFE-CO_2_/EtOH:H_2_O) to recover the medium-polarity fraction. Three levels of co-solvent (5, 10, and 15% *w*/*w*) were explored, and their effect on extraction yield, recovery of phenolic compounds (TPC and TFC), and antioxidant activity (DPPH scavenging) were evaluated. The results obtained are summarized in Table 4. SFE-CO_2_/EtOH:H_2_O enabled the recovery of medium-polarity extracts, and the extraction yield increased with the co-solvent percentage, reaching a maximum value at 15% (*w*/*w*) EtOH:H_2_O. This behavior can be attributed to the role of ethanol in increasing the polarity of the supercritical phase, thereby enhancing the solubility and mass transfer of medium-polarity compounds. Furthermore, ethanol likely disrupts solute–matrix interactions, as its hydroxyl groups can form hydrogen bonds with both the IGP matrix and the target solutes, facilitating their release [24].

The use of co-solvent significantly improves the recovery of phenolic compounds. Compared to stage 1, the TPC in SFE-CO_2_/EtOH:H_2_O extracts was up to 13 times higher than that observed in the extract obtained under SFE-CO_2optimal_. In addition, other nutraceuticals, such as flavonoids, were also recovered, enhancing the functional properties of the medium-polarity fraction. Consistent with the trend observed for extraction yield results, the TPC and TFC increased as the EtOH:H_2_O percentage was raised. The higher values were observed at 15% of co-solvent (105.35 ± 5.25 mg GAE/g extract and 82.12 ± 3.78 mg QE/g extract, respectively). The TPC and TFC values observed in the present work are higher than those reported in previous studies that employed SFE-CO_2_ with a co-solvent for the extraction of grape pomaces [30,31]. Otero-Pareja et al. [31] observed a maximum TPC of 70 mg GAE/g in extracts obtained by SFE-CO_2_ added with 20% (*w*/*w*) of ethanol as co-solvent from pomaces of various grape cultivars (Petit Verdot, Tintilla, Syrah, Cabernet, Merlot, Tempranillo). These results suggest that the defeating step using on the IGP improved the selectivity of SFE-CO_2_/EtOH:H_2_O process, thereby enhancing the efficiency of the phenolic compounds recovery.

All medium-polarity extracts exhibited DPPH scavenging activity, and this property was also dependent on EtOH:H_2_O percentage. The DPPH scavenging data were correlated with TPC and TFC results using Pearson correlation analysis, high correlations were observed between TPC/DPPH scavenging (R^2^ = 0.97) and between TFC/DPPH scavenging (R^2^ = 0.99). These results indicate that phenolic compounds are mainly responsible for the observed antioxidant activity. The medium-polarity fraction was selectively recovered from defatted IGP using SFE-CO_2_/EtOH:H_2_O. A co-solvent percentage of 15% was optimal for obtaining extracts with the highest content of phenolic nutraceuticals and the most potent DPPH scavenging activity.

### 3.3. Stage 3: Recovery of Phenolic-Rich Fraction

Another defatted IGP sample was independently prepared and subjected to recover its phenolic-rich fraction using PLE-EtOH:H_2_O. A RCCD was employed to explore the effects of temperature and solvent composition on the response variables (extraction, yield, TPC, TFC, TAC, and TCTC), and subsequently to determine the optimal conditions for recovering the phenolic-rich fraction. Table 2 summarizes the overall results obtained from stage 3. High temperature provides the highest extraction yields, with values ranging from 21.02% to 25.14%. Specifically, extraction yields at temperatures above 100 °C were up to 15 times higher than those achieved at lower temperatures (e.g., Run 2) and up to 4 times greater than those obtained at intermediate temperatures (e.g., Runs 9–12, central point). Note that this trend was consistent across all ethanol percentages investigated. A similar behavior was observed for the recovery of all phenolic compounds, with their highest recorded at temperatures over 100 °C.

**Figure 4:**
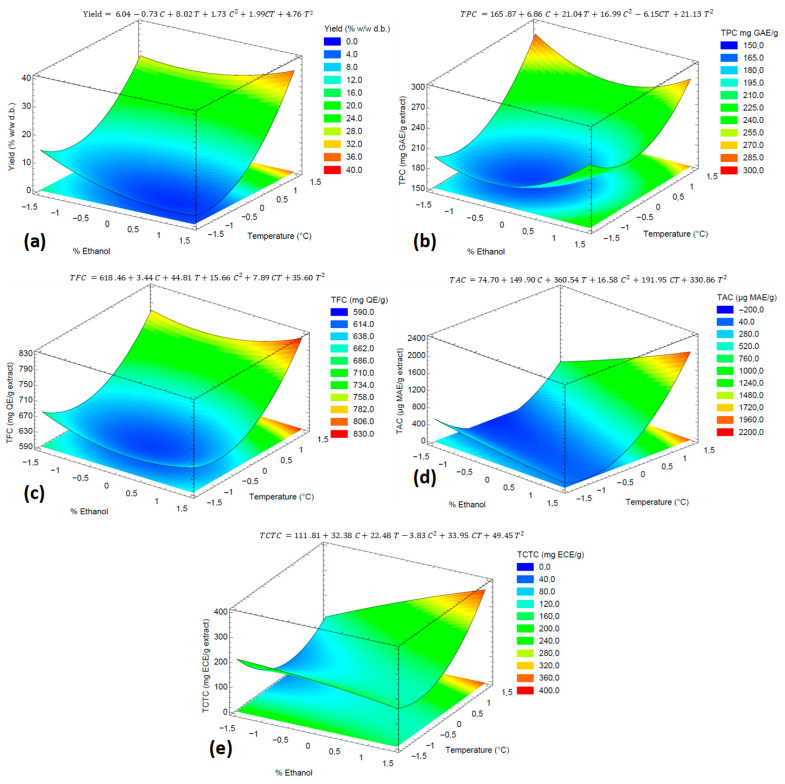
Response surface plots of the effects of PLE parameters on (**a**) extraction yield, (**b**) TPC, (**c**) TFC, (**d**) TAC, and (**e**) TCTC. Ethanol percentage and temperature coded levels are described in Table 2.

The dataset results (Table 2) were fitted by RSM coupled with multiple regression to describe the effect of the PLE variables on the process yield and phenolic contents of the extracts. Table S1 presents the analysis of variance (ANOVA) results for the second-order model fit and the effect of the extraction parameters. In addition, the second-order equations for each response variable are present in Figure 4. All mathematical models showed no lack of fit (*p* > 0.05). Furthermore, the R^2^ and adj-R^2^ coefficients were greater than 90.80% and 82.28%, respectively, suggesting a high predictability ability of the models. According to the Pareto’s analysis (see Figure S2), the temperature (both linear and quadratic coefficients) had the most significant effect (*p* < 0.001) on the extraction yield. This effect is evident in the response surface plot shown in Figure 4a: the yield increased with temperature across all ethanol percentages, with the most pronounced change occurring above 80 °C. Similar results were observed for the recovery of phenolic compounds, with the exception of condensed tannins: the TFC and TAC were influenced solely by temperature (*p* < 0.01); however, the solvent composition demonstrated no significant influence. A similar trend was observed for TPC, although in this case the quadratic coefficient of the solvent composition also had a significant effect. Figure 4b–d shows that TPC, TFC, and TAC increase rapidly above 80 °C, reaching their maximum values around 110 °C.

The results demonstrated that PLE-EtOH:H_2_O, employing high temperatures (near 108.3 °C) and ethanol percentage (above 50%), efficiently recovered phenolic compounds from the defatted IGP and provided a high extraction yield. These results can be explained by the fact that high temperatures disrupt solute–matrix interactions, promoting the desorption and release of solutes. Furthermore, high temperature enhances mass transfer and increases solute solubility in the EtOH:H_2_O mixture [18]. On the other hand, the increase in ethanol concentration contributed to the recovery of phenolic compounds; this is likely due to the enhanced solubility of phenolics and the amphipathic nature of ethanol, which facilitates interactions with compounds of varying polarities. Notably, this behavior was more pronounced in the recovery of condensed tannins in the present study. The observed effect of temperature on phenolics recovery has been previously reported for PLE with EtOH:H_2_O (50% *w*/*w*) applied to other grape pomaces [23]. In these studies, the optimal results were observed at temperatures ranging from 100 °C to 120 °C.

Table 2 shows that all phenolic-rich extracts exhibited DPPH scavenging activity; as expected, the extracts with the highest phenolic content demonstrated the greatest antioxidant activity. The highest DPPH scavenging activity was observed in the extracts obtained at 100 °C with 60% ethanol and at 108.3 °C with 50% ethanol, with values of 130.78 ± 1.93 and 130.40 ± 1.56 mmol TE/g extract, respectively. This activity is higher than that reported for extracts obtained from other pomaces (e.g., Cabernet Sauvignon, Merlot, and Bordeaux) by conventional extraction methods [32,33]. Pearson correlation analysis indicated a strong correlation between DPPH scavenging activity and yield (R^2^ = 0.93), TPC (R^2^ = 0.95), and TFC (R^2^ = 0.90). In contrast, no significant correlation was found for TAC or TCTC with DPPH scavenging activity.

The optimal conditions for PLE-EtOH:H_2_O stage were established using MRO. The optimal PLE parameters (PLE_optimal_) were an ethanol concentration of 58.91% and a temperature of 107.98 °C, with a desirability function of 0.98. The MRO model predicted optimal values of 27.60% for extraction yield, 238.56 mg GAE/g extract for TPC, 758.09 mg QE/g extract for TFC, 1510 µg MAE/g extract for TAC, and 264.36 mg ECE/g extract for TCTC. Experimental verification under PLE_optimal_ confirmed these predictions: yield of 27.10 ± 0.90%, 236.46 ± 3.55 mg GAE/g, TFC of 757.18 ± 6.25 mg QE/g, TAC of 1508 ± 3.14 µg MAE/g, and TCTC of 258.39 ± 3.78 mg ECE/g. To summarize stage 3, PLE-EtOH:H_2_O under optimal conditions successfully recovered a phenolic-rich fraction from IGP, containing diverse bioactive compounds including phenolic acids, flavonoids, anthocyanins, and condensed tannins.

### 3.4. Overall Process Integration and Efficiency

The sequential application of SFE-CO_2_, SFE-CO_2_/EtOH:H_2_O, and PLE-EtOH:H_2_O enabled comprehensive valorization of Isabella grape pomace by recovering distinct nutraceutical fractions based on their polarity. The integrated process achieved a total cumulative extraction yield and TPC of 41.08% and 349.89 mg GAE/g extract, respectively (sum of optimal yields from all stages), demonstrating high efficiency in biomass utilization. More importantly, the sequential approach allowed for the fractionation of bioactive compounds according to their chemical properties: lipophilic compounds (low-polarity phenolics, PUFAs, and tocopherols) in stage 1, medium-polarity phenolics (flavonoids and flavones) in stage 2, and highly polar phenolic compounds (flavonoids, anthocyanins, and condensed tannins) in stage 3.

The defatting step in stage 1 proved crucial for enhancing the recovery efficiency in subsequent stages. By removing the lipophilic fraction, the matrix porosity increased and solute–matrix interactions were disrupted easily, facilitating improved access of solvents to phenolic compounds in stages 2 and 3. This explains the superior TPC values obtained in stage 2 (up to 105.35 mg GAE/g extract) compared to conventional single-step SFE with co-solvent reported in the literature [30,31]. The lipophilic fraction showed a composition rich in linoleic acid (65.5%) and α-tocopherol (107.2 mg/kg), comparable to premium edible oils. This fraction possesses significant potential as a functional ingredient in nutraceutical formulations, given the well-established health benefits of PUFAs and vitamin E [8,9].

The medium-polarity fraction from stage 2 exhibited remarkably phenolic content (105.35 mg GAE/g extract) and high DPPH radical scavenging (0.18 mmol TE/g extract). On other hand, the phenolic-rich fraction obtained Via PLE-EtOH:H_2_O demonstrated exceptional nutraceutical potential, with TPC values (236.46 mg GAE/g extract) exceeding those reported for most grape pomace extracts in the literature [32,33]. The high concentrations of flavonoids (757.18 mg QE/g extract) and anthocyanins (1508 μg MAE/g extract) suggest promising applications in functional foods and natural colorants. Finally, the strong correlation between phenolic content and antioxidant activity across all fractions underscores the central role of phenolic compounds in the bioactivity of IGP extracts. This relationship is particularly relevant for potential applications in food preservation and health promotion.

## 4. Conclusions

The present study demonstrates that Isabella grape pomace, currently an underutilized waste stream in the Colombian wine industry, represents a valuable source of diverse nutraceutical compounds. The sequential green extraction approach developed herein aligns with circular economy principles and offers a sustainable alternative to conventional waste management practices. In stage 1, SFE-CO_2optimal_ (31.7 MPa, 58.9 °C) enabled efficient recovery of a lipophilic fraction rich in linoleic acid and α-tocopherol. Meanwhile, stage 2 SFE-CO_2_ added with 15% of ethanol as co-solvent effectively extracted medium-polarity phenolics, achieving high TPC values and demonstrating strong antioxidant activity. Finally, stage 3, PLE under optimal extraction condition (58.91% EtOH, 107.98 °C) produced a phenolic-rich fraction with promising nutraceutical properties: TPC (236.46 mg GAE/g extract), TFC (757.18 mg QE/g extract), TAC (1508 μg MAE/g extract), and potent antioxidant capacity (130.40 mmol TE/g extract). While this approach aligns with circular economy principles and offers a sustainable alternative to conventional waste disposal, certain practical limitations (e.g., overall processing time, SFE, and PLE equipment entail higher capital and operational costs) should be addressed and explored in order to establish the processes viability. Future research should focus on scaling up the process and developing specific applications in functional foods and nutraceutical products.

## Figures and Tables

**Table 1:** Experimental design and results of extraction yield and total phenolic content of the non-polar extracts obtained in stage 1.

Run	Coded Variables ^a^		Pressure (MPa)	Temperature (°C)	Extraction Yield (% *w*/*w* d.b.)	TPC ^b^ (mg GAE/g Extract)
	*P*	*T*				
1	−1	−1	20	40	6.24	5.64 ± 0.26 ^c^
2	+1	−1	30	40	6.41	3.33 ± 0.08 ^e^
3	−1	+1	20	60	3.72	3.00 ± 0.14 ^f^
4	+1	+1	30	60	6.60	6.48 ± 0.07 ^b^
5	0	−1.414	25	35.9	5.94	2.98 ± 0.15
6	0	+1.414	25	64.1	6.22	2.82 ± 0.05
7	−1.414	0	11.9	50	4.68	4.19 ± 0.02 ^d^
8	+1.414	0	32.1	50	5.65	7.05 ± 0.46 ^a^
9	0	0	25	50	4.69	3.05 ± 0.25 ^ef^
10	0	0	25	50	4.72	3.05 ± 0.25 ^ef^
11	0	0	25	50	4.74	3.05 ± 0.25 ^ef^
12	0	0	25	50	4.72	3.05 ± 0.25 ^ef^

^a^ Temperature (*T*); Pressure (*P*). ^b^ TPC columns show the mean ± standard deviation (n = 3). Central point was replicated four times (Run 9–12); TPC values for central point correspond to average (n = 4). Means in columns followed by the same letter are not statistically different according to ANOVA test.

**Table 2:** Experimental design and results of extraction yield, TPC, TFC, TAC, TCTC, and antioxidant activity of the phenolic-rich extracts obtained in the stage 3.

Run	Independent Variables		Response Variables ^a^					
	Ethanol	Temperature	Yield	TPC	TFC	TAC	TCTC	DPPH ^b^
	%EtOH	(°C)	% *w*/*w* d.b.	mg GAE/g	mg QE/g	µg MAE/g	mg ECE/g	mmol TE/g
1	40 (−1)	60 (−1)	7.50	166.26 ± 1.90 ^f^	614.87 ± 3.27 ^f^	40.26 ± 0.18 ^i^	137.63 ± 3.33 ^d^	9.15 ± 0.62 ^e^
2	60 (+1)	60 (−1)	1.63	196.29 ± 3.04 ^c^	590.36 ± 10.35 ^g^	68.51 ± 0.56 ^h^	117.05 ± 5.51 ^f^	3.07 ± 0.14 ^h^
3	40 (−1)	100 (+1)	21.02	229.54 ± 1.90 ^a^	703.97 ± 1.64 ^c^	169.19 ± 0.35 ^e^	131.75 ± 1.66 ^e^	113.89 ± 0.87 ^b^
4	60 (+1)	100 (+1)	23.11	234.96 ± 2.04 ^a^	711.04 ± 3.31 ^b^	965.20 ± 0.64 ^b^	246.97 ± 7.73 ^a^	130.78 ± 1.93 ^a^
5	50 (0)	57.1 (−1.414)	4.50	181.88 ± 1.33 ^e^	651.76 ± 5.70 ^e^	190.64 ± 0.58 ^d^	190.06 ± 9.27 ^b^	6.19 ± 0.26 ^g^
6	50 (0)	108.3 (+1.414)	25.14	228.85 ± 4.79 ^a^	756.94 ± 14.81 ^a^	1504.89 ± 2.43 ^a^	249.56 ± 1.67 ^a^	130.40 ± 1.56 ^a^
7	35.9 (−1.414)	80 (0)	9.50	190.21 ± 0.96 ^d^	648.54 ± 16.51 ^e^	86.60 ± 0.31 ^f^	45.09 ± 5.04 ^g^	15.89 ± 0.40 ^d^
8	64.1 (+1.414)	80 (0)	8.03	203.97 ± 6.82 ^b^	680.30 ± 5.03 ^d^	351.75 ± 6.01 ^c^	161.37 ± 7.54 ^c^	18.66 ± 0.34 ^c^
9	50 (0)	80 (0)	6.18	165.87 ± 7.66 ^f^	618.46 ± 9.50 ^f^	74.67 ± 3.22 ^g^	111.81 ± 17.57 ^f^	7.23 ± 0.51 ^f^
10	50 (0)	80 (0)	6.66					
11	50 (0)	80 (0)	5.15					
12	50 (0)	80 (0)	6.20					

^a^ All responses data are shown as the mean ± standard deviation (n = 3). Central point was replicated four times (Run 9–12), TPC, TFC, TAC, TCTC and DPPH scavenging values for central point correspond to average (n = 4). Means in columns followed by the same letter are not statistically different according to ANOVA test. ^b^ DPPH scavenging activity.

**Table 3:** Fatty acids composition and tocopherols content of lipophilic fraction obtained at optimal SFE-CO_2_ conditions.

Fatty Acids (%) ^a^	
Linoleic acid	65.5
Oleic acid	25.3
Palmitic acid	7
Stearic	1.2
Others (linolenic, araquidonic)	>1
**Tocopherols (mg kg^−1^) ^b^**	
α-Tocopherol	107.2 ± 0.14
β-Tocopherol	0.95 ± 0.01
γ-Tocopherol	14.2 ± 0.26
δ-Tocopherol	0.65 ± 0.01

^a^ Relative percentage of each fatty acid identified in the total FAME profile. ^b^ Results are presents as the mean ± standard deviation (n = 3).

**Table 4:** Extraction yield, phenolic composition, and antioxidant activity of the medium-polarity extracts obtained from defatted IGP in stage 2.

Co-Solvent Level (% *w*/*w*)	Extraction Yield (% *w*/*w* d.b.)	TPC (mg GAE/g Extract)	TFC (mg QE/g Extract)	DPPH scavenging (mmol TE/g Extract)
5	4.03 ± 0.15 ^c^	70.11 ± 3.31 ^c^	47.36 ± 1.04 ^c^	0.08 ± 0.01 ^c^
10	5.75 ± 0.08 ^b^	82.78 ± 5.21 ^b^	62.80 ± 1.73 ^b^	0.12 ±0.02 ^b^
15	7.03 ± 0.27 ^a^	105.35 ± 5.25 ^a^	82.12 ± 3.78 ^a^	0.18 ± 0.01 ^a^

All data are shown as the mean ± standard deviation (n = 3). Means in columns followed by the same letter are not statistically different according to ANOVA test.

## Data Availability

All data used for the analyses carried out in this review are included in the article and Supplementary Materials. Any additional data will be available upon request to the authors.

## Supplementary Materials

The following supporting information can be downloaded at https://www.mdpi.com/article/10.3390/foods15010054/s1, Figure S1. Pareto Chart for (a) extraction yield and (b) TPC. A: Pressure; B: Temperature. Figure S2. Pareto Chart for (a) extraction yield, (b) TPC, (c) TFC, (d) TAC, and (e) TCTC. A: Pressure; B: Temperature. Table S1. Analysis of variance of regression coefficients and statistical indicators of second order model for extraction yield, TPC, TFC, TAC, TCTC.

## Abbreviations

The following abbreviations are used in this manuscript:

IGP	Isabella grape pomace
PUFAs	Polyunsaturated fatty acids
GRAS	Generally Recognized as Safe
SFE	Supercritical fluid extraction
PLE	Pressurized liquid extraction
CO_2_	Carbon dioxide
RCCD	Rotable central composite design
ANOVA	Analysis of variance
TFC	Total flavonoid content
TPC	Total phenolic content
TCTC	Total condensed tannins content
TAC	Total anthocyanin content
RSM	Response surface model
