# Supplementary material for: Valorization of Isabella Grape (Vitis labrusca L.) Pomace Through the Recovery of Nutraceuticals by Sequential Green Extraction Technologies
Source: Foods. 2025 Dec 24;15(1):54. doi: 10.3390/foods15010054 (PMC12785773; doi:10.3390/foods15010054)
Supplement: Supplementary file 1 [file foods-15-00054-s001.zip › foods-4015465-supplementary.pdf]

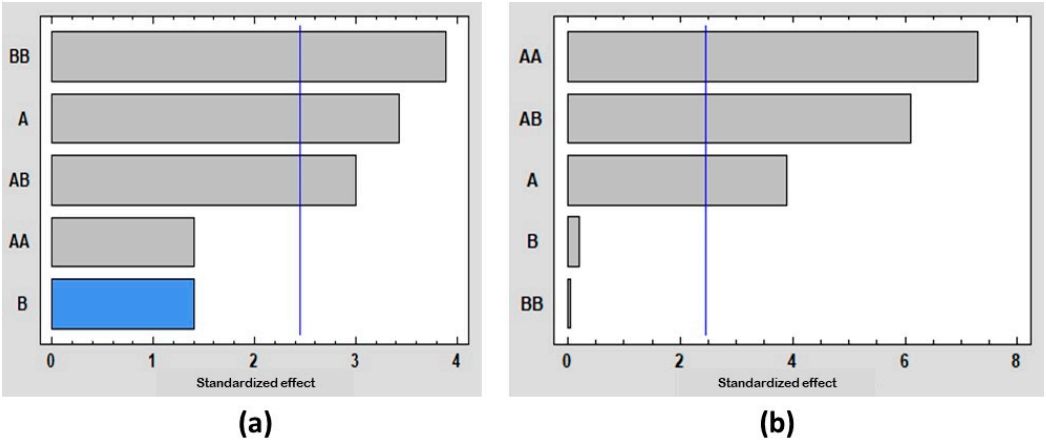


**Figure S1.** Pareto Chart for (a) extraction yield and (b) TPC. A: Pressure; B: Temperature.

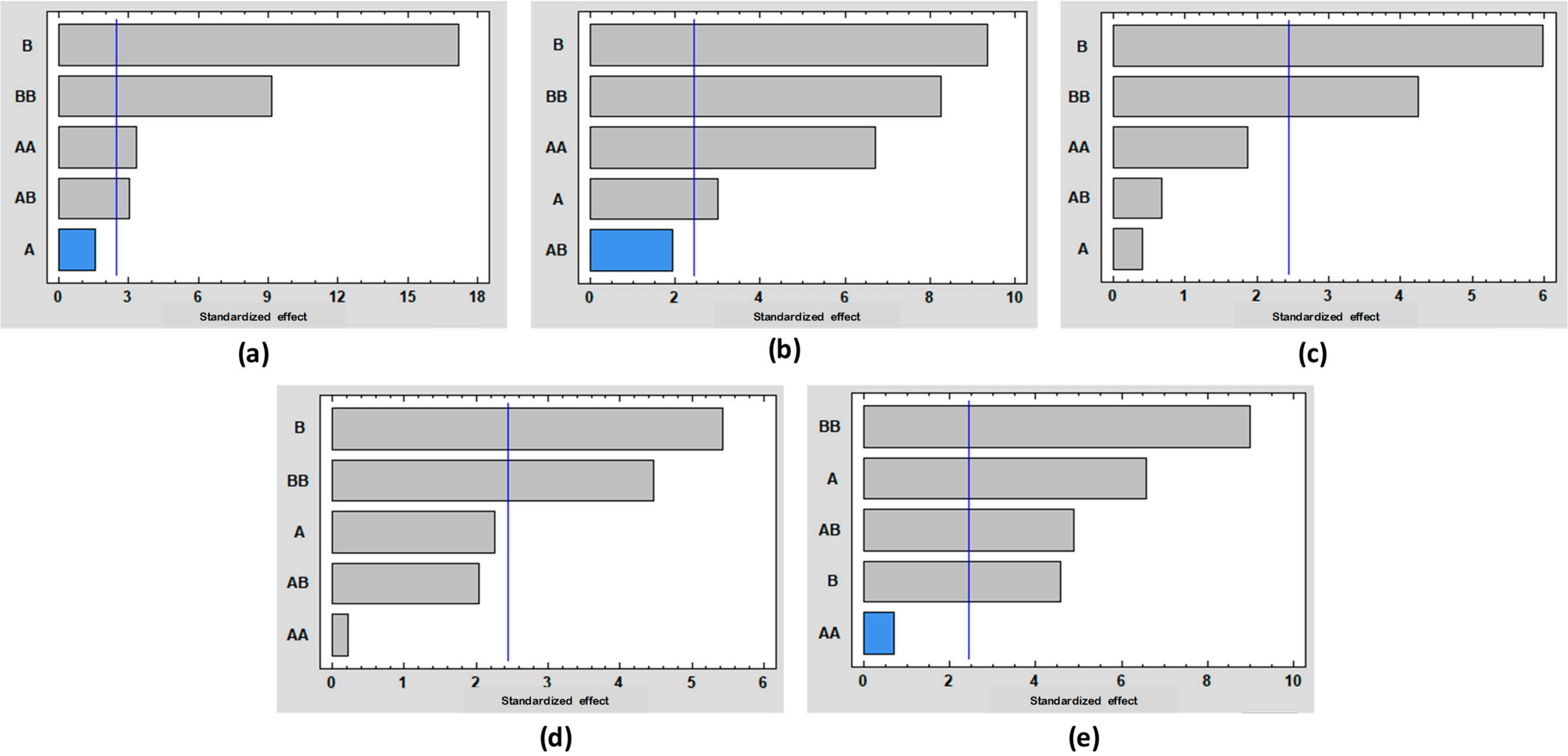


**Figure S2.** Pareto Chart for (a) extraction yield, (b) TPC, (c) TFC, (d) TAC, and (e) TCTC. A: Pressure; B: Temperature.

**Table S1.** Analysis of variance of regression coefficients and statistical indicators of second order model for extraction yield, TPC, TFC, TAC, TCTC.

| | **Extraction yield** | | **TPC** | | **TFC** | | **TAC** | | **TCTC** | |
|---|---|---|---|---|---|---|---|---|---|---|
| | **Coefficient** | ***p*-value** | **Coefficient** | ***p*-value** | **Coefficient** | ***p*-value** | **Coefficient** | ***p*-value** | **Coefficient** | ***p*-value** |
| $C$ [a] | -0,73 | 0,1674 | 6,86 | 0,0240* | 3,44 | 0,6604 | 149.90 | 0,0645 | 32,38 | 0,0006** |
| $T$ [b] | 8,02 | <0,0001** | 21,04 | 0,0001** | 44,81 | 0,0010** | 360.53 | 0,0016** | 22,48 | 0,0041** |
| $C^2$ | 1,73 | 0,0157* | 16,99 | 0,0006** | 15,66 | 0,1097 | 016.58 | 0,8305 | -3,83 | 0,5180 |
| $CT$ | 1,99 | 0,0235* | -6,15 | 0,1058 | 7,89 | 0,4829 | 191.95 | 0,0867 | 33,95 | 0,0030** |
| $T^2$ | 4,76 | 0,0001** | 21,13 | 0,0002** | 35,60 | 0,0053** | 330.85 | 0,0043** | 49,45 | 0,0001** |
| Lack of fit | ($p$ = 0.674) | | ($p$ = 0.117) | | ($p$ = 0.881) | | ($p$ = 0.778) | | ($p$ = 0.862) | |
| $R^2$ | 98.50% | | 96.96% | | 94.34% | | 90.80% | | 96.60% | |
| Adj-$R^2$ | 97.25% | | 94.45% | | 82.28% | | 83.13% | | 93.75% | |
| Standard error | 1.32 | | 0.47 | | 0.408 | | 0.187 | | 0.123 | |

[a] Solvent composition

[b] Temperature

**highly significant ($p < 0.01$)

*significant (0.01 < $p$ < 0.05
